# Prolonged Febrile Seizures Impair Synaptic Plasticity and Alter Developmental Pattern of Glial Fibrillary Acidic Protein (GFAP)-Immunoreactive Astrocytes in the Hippocampus of Young Rats

**DOI:** 10.3390/ijms232012224

**Published:** 2022-10-13

**Authors:** Alexandra V. Griflyuk, Tatyana Y. Postnikova, Aleksey V. Zaitsev

**Affiliations:** Sechenov Institute of Evolutionary Physiology and Biochemistry of RAS, 44, Toreza Prospekt, 194223 Saint Petersburg, Russia

**Keywords:** febrile seizures, hyperthermia, long-term potentiation, astrocyte, hippocampus

## Abstract

Prolonged neonatal febrile seizures (FSs) often lead to cognitive decline and increased risk of psychopathology in adulthood. However, the neurobiological mechanisms underlying the long-term adverse effects of FSs remain unclear. In this study, we exposed rat pups to hyperthermia and induced FSs lasting at least 15 min. We investigated the short-term (one day) and delayed (11–13 and 41–45 days) effects of FSs on some parameters of morphological and functional maturation in the hippocampus. We noticed that FSs altered the developmental pattern of glial fibrillary acidic protein (GFAP) immunoreactivity. In rats aged 21–23 days, GFAP-positive astrocytes covered a smaller area, and their morphological characteristics resembled those of rats at 11 days of age. In post-FS rats, the magnitude of long-term synaptic potentiation was reduced compared to control animals of the same age. Applying the gliotransmitter D-serine, an agonist of the glycine site of NMDA receptors, restored LTP to control values. A decrease in LTP amplitude was correlated with impaired spatial learning and memory in the Barnes maze task in post-FS rats. Our data suggest that impaired neuron–glia interactions may be an essential mechanism of the adverse effects of FS on the developing brain.

## 1. Introduction

Early age is the most important critical developmental period. The brain is being shaped during this period, and early-life adversity may disrupt normal brain development [1,2]. Such adversity can be trauma [3], malnutrition [4], infections, febrile seizures (FSs) [5,6,7,8], and others, which lead to cognitive decline and an increased risk of psychopathology in adulthood. In addition, stress experienced at an early age leads to a change in the regulation of the hypothalamic–pituitary–adrenal axis, which in adulthood is manifested by an increased response to stressful stimuli [9,10,11] and may promote vulnerability to epileptogenesis [12].

FSs are among the most common neurological disorders in children aged between 3 months and 5 years, with a peak incidence in the second year of life, and are typically caused by infectious diseases with high fever [13,14]. FSs are classified as simple and complex (recurrent or prolonged) depending on the duration and the presence of recurrent episodes. Simple FSs last less than 15 min, and the attacks occur no more than once in 24 h. Complex FSs include seizures lasting more than 15 min or recurring episodes during the day [15,16]. Usually, simple FSs do not have long-term adverse effects, while prolonged FSs more often lead to various psychopathologies, including learning and memory deficits [17,18,19,20,21].

Despite numerous studies, the neurobiological mechanisms underlying the pathophysiology of prolonged FSs remain unclear. Epileptic seizures can impair brain activity due to synaptic dysfunction: altering the probability of mediator release, the composition of postsynaptic receptor subunits, and the properties of astrocytes and microglia [22]. Research on this topic is difficult in humans because of many limitations, so animal models, especially rat models, have been used to study the mechanisms underlying the long-term adverse effects of FSs [5,23,24]. In these models, febrile seizures are most often induced in 9–11-day-old (P (postnatal) 9–P11) rat pups with a heated stream of air [5,23,24,25]. Among various brain structures, the hippocampus has been extensively studied because of its key role in cognitive functions [26,27].

According to current concepts, synaptic transmission and plasticity are determined not only by neurons but also by the surrounding glial cells [28]. The properties of long-term synaptic plasticity, which is a cellular mechanism of memory, may be an indicator of hippocampal maturation and functional activity. The magnitude of long-term synaptic potentiation (LTP) increases after birth, becoming similar to the LTP of adult animals by the end of the second week of life [29].

During the first two weeks of postnatal development, cell networks undergo morphological and functional maturation in the rodent brain. This relates not only to neuronal maturation, which includes the dramatic growth of dendrites and the formation of synapses, but also includes the differentiation and maturation of astrocytes and the astrocytic network [30]. Most astrocytes are generated during the second postnatal week [31,32]; astrocytes at P11 appeared to have large cell bodies and short branches in CA1 and CA3 of the hippocampus and the dentate gyrus. In older rats, astrocytes appeared to have a smaller cell bodies with longer processes with thin and ramified branches [31]. Astrocyte proliferation decreases sharply after two postnatal weeks, although a moderate increase in the number of astrocytes is observed until 1 month of age [31].

In our previous study, we found that febrile seizures in P10 cause a significant attenuation of LTP induction in the hippocampus in rats at P21 [25]. We also found that the addition of D-serine, an NMDA receptor glycine site agonist, restored LTP, which may indicate impaired neuron–glia interactions. In this work, we aimed to analyze how the morphological maturation of astrocytes as well as the properties of synaptic plasticity in the hippocampus in rats at P12, P21–23, and P51–55 after FSs at P10 are affected by FSs.

## 2. Results

### 2.1. Febrile Seizures Impair Long-Term Synaptic Plasticity

Experimental FSs were induced in rat pups at P10. The pups were heated with a warm airflow (46 °C) for 30 min. The development and course of FSs in most animals were stereotypic: during the first 10 min, body temperature rose to 39 °C, facial automatisms were observed, often accompanied by unilateral body flexion. Then, there were myoclonic twitches of the hind limbs, followed by clonic convulsions. Body temperature (rectal) was measured every two minutes during seizures. If the body temperature was above 41 °C, the rats were transferred to a cool surface until the body temperature returned to 39 °C or less. The rats were then transferred back to the chamber. Only animals with FSs that lasted at least 15 min were included in the study (*N* = 53).

**Figure 1:**
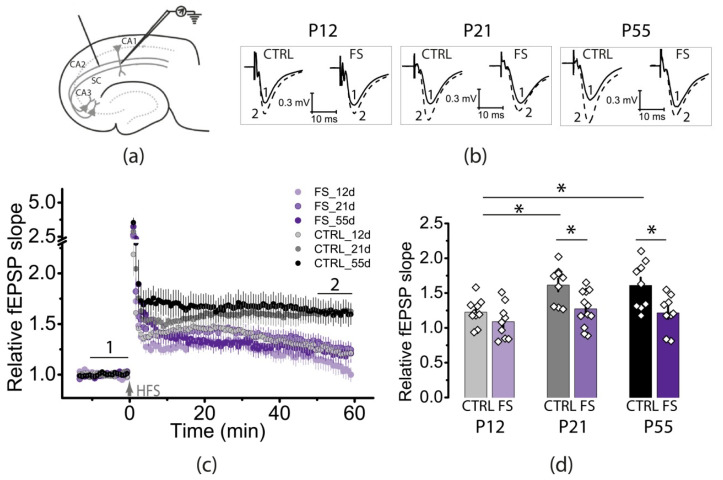
Febrile seizures impair long-term synaptic plasticity. (**a**) Schema showing the positions of the electrodes in the hippocampus. CA1, CA2, CA3—hippocampal areas, SC—Schaffer collaterals. (**b**) Representative examples of field excitatory postsynaptic potential (fEPSP) before induction (1) and 50–60 min after high-frequency stimulation (HFS) (2). (**c**) Diagram showing changes in the value of the normalized slope of fEPSP in control (CTRL) and experimental (FS) animals of different ages (P12, P21, and P55) after LTP induction (arrow indicates the time of high-frequency stimulation (HFS). (**d**) Diagram illustrating the differences in LTP between the control (CTRL) and experimental (FS) animals. Each diamond represents a value obtained for each slice. All data are presented as means ± standard error of the mean. Asterisks indicate significant differences between groups according to Tukey’s post hoc tests: * *p* < 0.05.

We examined LTP at CA3-CA1 synapses in the hippocampus of rats of different ages (P12, P21, and P55) in the control group and rats exposed to FSs at P10 (Figure 1). LTP was induced with a high-frequency stimulation (HFS) protocol. A two-way ANOVA revealed significant effects of age and FS on LTP magnitude (factor 1 “Age”: F_2,52_ = 6.9, *p* < 0.01 and factor 2 “FS”: F_1,52_ = 17.6, *p* < 0.001). However, there was no interaction of the factors (Age × FS: F_2,52_ = 1.27, *p* = 0.29).

We noticed age-related dynamics of changes in the LTP values in control animals. The LTP values in P12 animals were lower (1.23 ± 0.06, *N* = 7 rats, *n* = 10 slices) compared to P21–23 rats (1.61 ± 0.09, *N* = 7, *n* = 9, *p* < 0.05) and P51–55 animals (1.61 ± 0.11, *N* = 6, *n* = 9, *p* < 0.05). We also observed differences in the time course of LTP in control animals: in P12 animals, potentiation persisted for approximately 30 min after HFS then gradually decreased, whereas in P21 and P55 animals, potentiation persisted at a steadily high level for one hour after HFS. These results are consistent with those of other researchers [29].

The effect of FS on LTP magnitude was distinct only in the two older age groups. In P12 rats subjected to FSs, we observed an almost complete absence of synaptic potentiation after 1 h (1.09 ± 0.08, *N* = 5, *n* = 9), although this value did not differ statistically from that of control P12 rats (*p* = 0.87). It should also be noted that in the beginning the time course of LTP development practically coincided in both groups of P12 rats (Figure 1c), but later LTP stabilized at a relatively low level in healthy animals, and the FS group showed a tendency to further decrease synaptic potentiation.

The LTP values were significantly lower in P21 rats after FSs (1.27 ± 0.07, *N* = 6, *n* = 12, *p* < 0.05) and at P55 (1.22 ± 0.09, *N* = 6, *n* = 9, *p* < 0.05). Interestingly, in P21 animals after FS, the time course of LTP development was similar to that observed in younger animals. We can assume that FSs slowed down the functional maturation of the hippocampus.

### 2.2. The Co-Agonist of NMDARs, D-Serine, Restored Long-Term Synaptic Potentiation in Rats after Febrile Seizures

LTP induction in hippocampal CA3-CA1 synapses requires the activation of NMDA receptors [33]. Therefore, to determine whether the NMDA-receptor-dependent mechanism of LTP induction changed after FS, we induced LTP in the presence of the NMDA receptor channel blocker MK-801 (10 μM).

**Figure 2:**
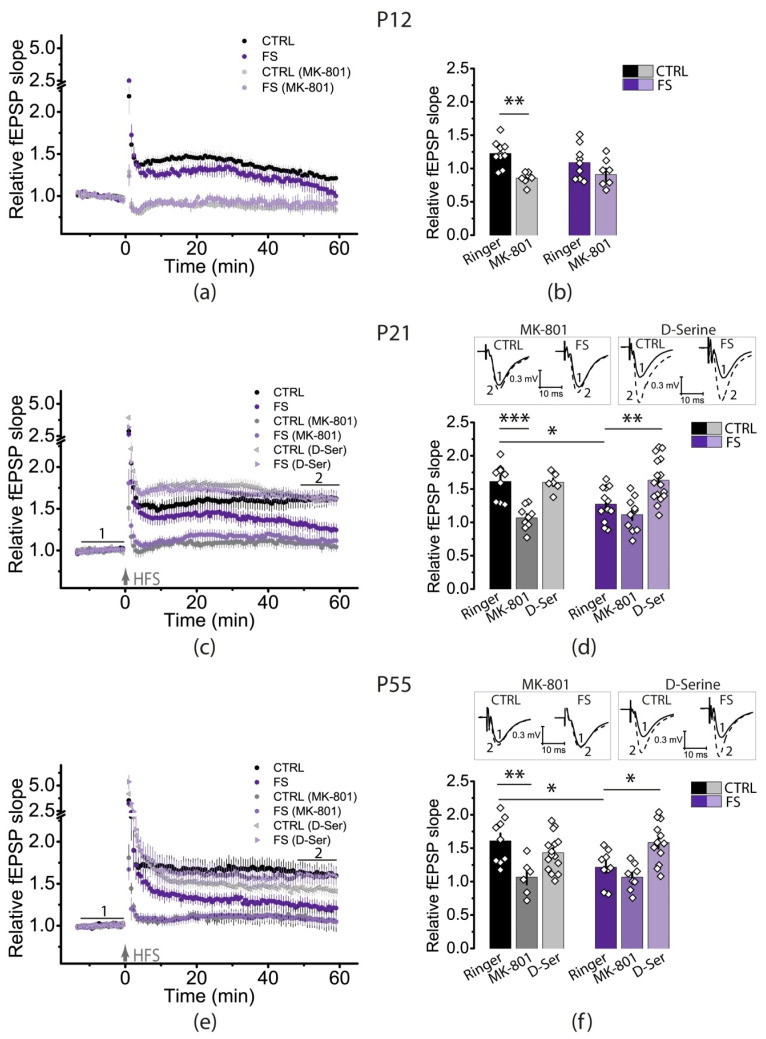
LTP induction at CA3-CA1 synapses in the hippocampus of rats of different ages after FSs was NMDAR-dependent, and a co-agonist of NMDARs, D-serine, restored LTP in rats at P21 and P55 after FSs. (**a**,**c**,**e**) Diagrams showing changes in the values of the normalized slope of fEPSP in control (CTRL) and experimental (FS) animals of different ages (P12, P21, and P55) after HFS in the presence of the NMDAR blocker MK-801 (10 μM) or in the presence of the NMDAR co-agonist D-serine (10 μM). (**b**,**d**,**f**) Diagrams illustrating the differences in LTP between the control (CTRL) and experimental (FS) animals of different ages in the presence of MK-801 or D-serine. Each diamond represents a value obtained for each slice. Representative examples of fEPSP before induction (1) and 50–60 min after HFS (2) in the presence of MK-801 or D-serine. All data are presented as means ± standard error of the mean. Asterisks indicate significant differences between groups according to Tukey’s post hoc tests: * *p* < 0.05, ** *p* < 0.01, *** *p* < 0.001.

In the control group, the application of MK-801 prevented the induction of LTP (Figure 2; P12: 0.86 ± 0.03, *N* = 6, *n* = 7, *p* < 0.01; P21: 1.07 ± 0.06, *N* = 8, *n* = 9, *p* < 0.001; P55: 1.07 ± 0.11, *N* = 6, *n* = 6, *p* < 0.01). In the FS group, the use of MK-801 also prevented LTP induction (P12: 0.91 ± 0.09, *N* = 6, *n* = 7; P21: 1.12 ± 0.08, *N* = 9, *n* = 10; P55: 1.07 ± 0.07, *N* = 5, *n* = 8). Our results confirm that in FS-exposed rats the mechanism of LTP induction remains an NMDA-receptor-dependent process.

The decrease in LTP may be due to the attenuation of NMDA-receptor-dependent signaling. The activation of the NMDA receptors requires the binding of two agonists: glutamate to the GluN2 subunit and glycine to the glycine site of the GluN1 subunit [34]. In our previous work, we revealed that in P21 rats subjected to FS there is a rapid desensitization of NMDA currents due to insufficient activation of the glycine sites of NMDA receptors [25]. The application of D-serine, the glycine site agonist, allows the restoration of LTP to a control value in rats exposed to FS [25].

In the present study, we focused on investigating the effect of D-serine on LTP production in P21 and P55 animals, as there was a significant attenuation of plasticity at these ages. In control animals, D-serine did not affect the LTP magnitude (Figure 2; P21: 1.60 ± 0.06, *N* = 6, *n* = 6; P55: 1.43 ± 0.07, *N* = 7, *n* = 15). At the same time, in the FS groups, D-serine restored the LTP magnitude to control values (P21: 1.60 ± 0.06, *N* = 8, *n* = 16, *p* < 0.01; P55: 1.59 ± 0.09, *N* = 8, *n* = 12, *p* < 0.05). It has previously been shown that the calcium-dependent release of D-serine into the synaptic cleft by astrocytes may control LTP [28]. Since astrocytes are the main source of D-serine [35], we can assume that FSs lead to disorders of neuron–glia relationships.

### 2.3. FSs Decrease the Area Occupied by Astrocytes

Our results show that FSs slow down the functional maturation of the hippocampus, which is manifested by a decrease in the magnitude of LTP (Figure 1). Since one of the probable causes of LTP abnormalities could be a disruption of neuron–glia relationships, we decided to investigate the effect of FS on astrocyte development in the hippocampus.

**Figure 3:**
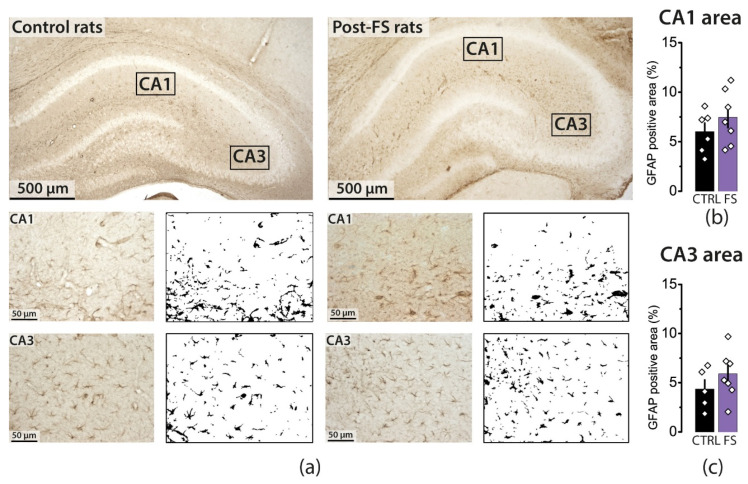
Animals at P12 after FSs showed no change in the area occupied by GFAP-positive objects. (**a**) Representative images of hippocampal sections (×5 magnification) and images of the stratum radiatum of the CA1 and CA3 fields (×40 magnification) and their black-and-white masks, which were obtained in the ImageJ program and used to count the area occupied by GFAP-positive objects. The averaged area occupied by GFAP-positive objects in the stratum radiatum of the CA1 (**b**) and CA3 (**c**) fields. The diamonds show the individual values for each rat.

Using immunohistochemical methods, we investigated the expression level of fibrillary acidic protein (GFAP) in the hippocampus of rats from the control and FS groups. We counted the area occupied by GFAP-positive objects in the *stratum radiatum* of the CA1 and CA3 fields. In animals at P12, there were no differences between groups (Figure 3; CA1: control—6.0 ± 0.9%, *N* = 6, FS—7.5 ± 1.0, *N* = 7, *t* = 1.04, *p* = 0.32; CA3: control—4.3 ± 0.9%, *N* = 5, FS—5.9 ± 1.0, *N* = 7, *t* = 1.07, *p* = 0.31). 

**Figure 4:**
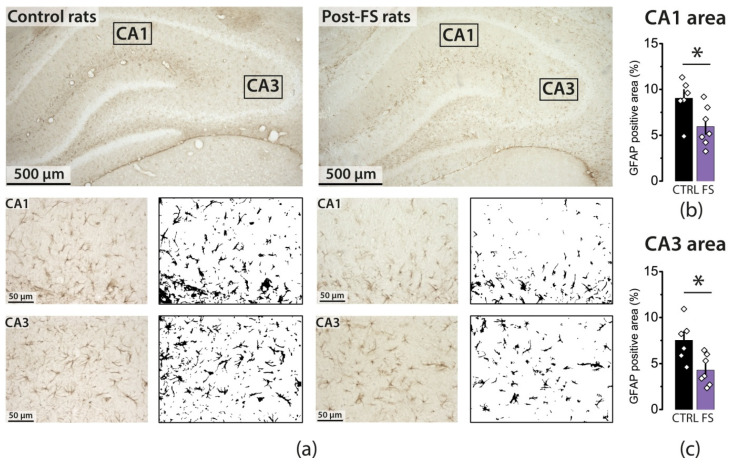
In animals at P21 after FSs, the area occupied by GFAP-positive objects was significantly less than in the control group. Animals at P12 after FSs showed no change in the area occupied by GFAP-positive objects. (**a**) Representative images of hippocampal sections (×5 magnification) and images of the stratum radiatum of the CA1 and CA3 fields (×40 magnification) and their black-and-white masks, which were obtained in the ImageJ program and used to count the area occupied by GFAP-positive objects. The averaged area occupied by GFAP-positive objects in the stratum radiatum of the CA1 (**b**) and CA3 (**c**) fields. The diamonds show the individual values for each rat. Asterisks indicate significant differences between groups according Student’s *t*-test: * *p* < 0.05.

However, in P21 animals exposed to FSs, the area occupied by GFAP-positive objects was significantly less than in the control group (Figure 4; CA1: control—9.0 ± 0.9%, *N* = 6, FS—6.0 ± 0.8%, *N* = 7, *t* = 2.52, *p* < 0.05; CA3: control—7.5 ± 0.9%, *N* = 6, FS—4.3 ± 0.6%, *N* = 7, *t* = 2.98, *p* < 0.05). Moreover, GFAP-positive astrocytes had rather short branches, and their morphological characteristics resembled those of rats at 11 days of age.

### 2.4. Cognitive Impairments

**Figure 5:**
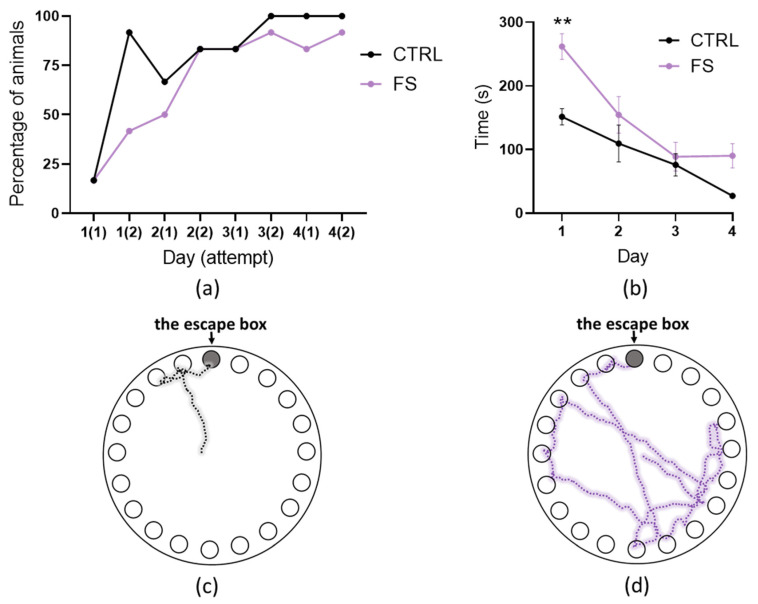
Training session (days 1 to 4) in the Barnes maze. (**a**) The percentage of control rats (CTRL) and rats after FSs (FS) that successfully completed each attempt. (**b**) Time spent searching for the escape box by control (CTRL) and experimental (FS) animals. Results of two-way ANOVA reported in the text. Asterisks indicate significant differences between groups according to Tukey’s post hoc tests: ** *p* < 0.01. Representative examples of the pathways of a control rat (**c**) and an FS rat (**d**) in the Barnes maze on day 4 (second attempt).

We observed prolonged impairments of long-term synaptic plasticity in rats after FSs. Therefore, we decided to use the Barnes maze to assess the spatial learning and memory in rats at P51–60 (Figure 5).

During the training sessions (days 1 to 4), the location of the escape box did not change. The rats were given two attempts for five minutes per day to find an escape box. If during this time the rat did not find the escape box, it was gently pushed and forced to go down into the box. In this case, we considered that the rat failed the attempt. Figure 5a shows the percentage of control rats and rats after FSs that successfully completed each attempt during training (*N* = 12 rats in each group). Rats in both groups learned to find the escape box, but more rats successfully completed the task in the control group than in the FS group at the beginning of the training. We also compared the average time the rats spent on both attempts per day (Figure 5b). A two-way ANOVA revealed a significant effect of learning (factor 1 “Day of training session”: F_3,66_ = 22; *p* < 0.001) and the role of the FSs at the tendency level (factor 2 “FS”: F_1,66_ = 3.8; *p* = 0.06). The interaction of the factors was not statistically significant (“FS” × “Day of training session”: F_3,66_ = 1.54; *p* = 0.21). We found a significant difference in the escape time only for the first day (Figure 5b). However, FS rats (Figure 5d) also tended to spend more time on the last day of training than control animals (Figure 5c).

**Figure 6:**
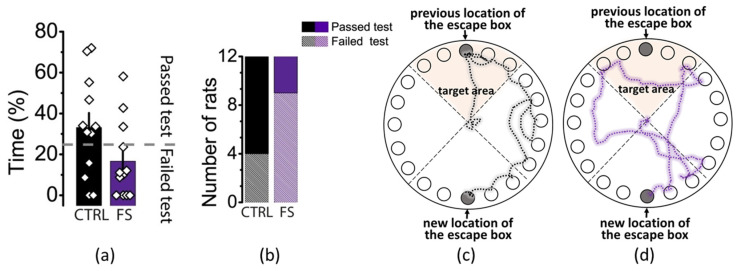
Test day (day 5). (**a**) Percentage of time spent by control (CTRL) and experimental (FS) rats in the target area. Each diamond represents an individual value. (**b**) Diagram showing the number of animals that passed and failed the test. Representative examples of the pathways of a control rat (**c**) and an FS rat (**d**) in the Barnes maze on the test day.

On the test day, the escape box was moved to the opposite side of the field (Figure 6). First, we assessed the time spent searching for the escape box and the distance traveled during this time. There were no differences in these parameters (time: control—119 ± 32 s, FS group—103 ± 28 s, *p* = 0.72; distance: control—5.1 ± 1.4 m, FS group—3.2 ± 0.7 m, *p* = 0.21). Next, we counted the percentage of time each animal spent in the target area (the sector of the field where the escape box was located during training) while searching for the new location of the escape box. Being in the target area for more than 25% of the time was considered a successful test result. In the control group, 8 of 12 animals passed the test, which was significantly more than among the FS animals (3 out of 12, *p* < 0.05, Fisher’s exact test). Thus, the spatial memory in FS animals was impaired compared to the control animals.

## 3. Discussion

In the present study, we investigated the characteristics of synaptic plasticity in the hippocampus of rats of different ages after prolonged FSs. We found that FSs cause a delay in the functional maturation of the hippocampus. In P21 rats, LTP characteristics resembled those of P11 rats. These changes were accompanied by slower morphological development in astrocytes. LTP impairment persisted until at least P55. At the behavioral level, the LTP deficit manifested as impaired spatial learning and memory in the Barnes maze task.

Here, we paid attention to the age-related dynamics of changes in the magnitude and pattern of LTP in the hippocampus of rats: in P12 animals, synaptic potentiation persisted for approximately 30 min after HFS then gradually decreased, whereas in P21–23 and P51–55 animals, potentiation persisted at a steadily high level for at least one hour after HFS. These findings are consistent with previous reports that rats less than two weeks old do not show robust LTP [36,37,38].

Liao and Malinow established that robust LTP can be induced in young animals if sufficient postsynaptic depolarization is provided during LTP induction [29]. Cao and Harris, using a different stimulus protocol, showed that, until P12, the late phase of LTP is not observed, and the magnitude of the late phase of LTP was enhanced as animals matured [39]. They noted that to induce the late phase of LTP in P10–11 animals it was necessary to use repeated stimulation. It was also shown that the calcium influx into the apical dendrites of CA1 pyramidal cells of the hippocampus, which is necessary for the induction of LTP, gradually increases during the second and third postnatal weeks in rats, and these dendritic calcium dynamics correlated with the maturation of synaptic plasticity [40]. In this study, we demonstrated that in 3-week-old rats after FSs the magnitude and pattern of LTP were similar to those observed in P12 animals, which suggests a delayed functional maturation of the hippocampus in FS rats.

The age-related dynamics of LTP development correlate with the maturation of astrocytes in the hippocampus. It has repeatedly been shown that astrocytes play a key role in the modulation of neuronal synaptic transmission, so the morphological and electrophysiological maturation of astrocytes affects their function. Our results are consistent with previous studies that showed that the GFAP-positive area increases significantly from the second to the third week of postnatal development [31]. We observed an increase in the size of GFAP-positive astrocytes and the branching level in the control group but not in the FS rat group. Thus, this observation also supports the idea of impaired functional maturation of the hippocampus. It can be assumed that the activation of astrocytes, in this case, could have a beneficial effect. An astrocyte-kinetic drug could be used for this purpose; for example, l-deprenyl, an irreversible inhibitor of monoamine oxidase type B, potentiates an astrocyte reaction associated with the increased secretion of trophic factors [41].

However, the obtained data should be interpreted with some caution since GFAP labeling only allows the visualization of primary, secondary, and occasionally tertiary branches of the astrocyte [42]. It should be also noted that during this period the electrophysiological maturation of astrocytes occurs as well, resulting in reduced voltage-gated ion conductances [43].

Periods of rapid development of any system are critical for their function formation and are most vulnerable to the action of injuring factors. Therefore, the consequences of febrile seizures can be diverse. It has been shown that FSs do not cause significant neuronal death in the hippocampus [25,27]. Moreover, experimental FSs in P10 rats increase the survival rate and structural integration of newborn dentate granule cells. Their dendrites develop faster, have more spines, and display increased spontaneous excitatory input [44,45]. FSs may cause long-term alterations in hippocampal functions. FSs trigger multiple changes in the expression of genes associated with heat, immune, and inflammation responses, the glutamate–glutamine cycle, myelination, and structural reorganization [46]. It is assumed that many signaling systems affecting synaptic transmission can be disrupted by FSs. For example, FSs may cause CB1 receptor upregulation, which in turn increases the depolarization-induced suppression of inhibition and leads to persistent limbic hyperexcitability [47]. The critical role of TRPV1 signaling was reported in a mouse model of FS, which led to the increased expression of proinflammatory cytokines (IL-1ß, IL-6, TNF-α, and HMGB1) [48]. It is well-established that these proinflammatory cytokines modulate synaptic transmission and plasticity [49,50,51].

There are relatively few experimental studies that have examined the effects of FSs on long-term synaptic plasticity. The data obtained in different laboratories are contradictory [25,52,53]. One study demonstrated that repeated FSs resulted in LTP attenuation and LTD facilitation in the CA1 hippocampal area [53]. In contrast, another study demonstrated an increase in LTP and a decrease in LTD in P44 rats that were exposed to FSs at P10 [52]. This experimental work unequivocally indicates an attenuation of LTP in P21 and P55 rats exposed to FSs. Discrepancies in the experimental results may be because FSs lead to the activation of several signaling systems affecting synaptic plasticity differently, which can lead to diverse outcomes. It should be noted that various early-onset seizure models in rodents usually result in long-term impairments of synaptic plasticity and cognitive function [54,55,56,57,58,59,60]. In a hypoxia model of seizures in P10 rats, increased LTP was detected instantly after the seizure [61]. However, reduced LTP was found 48–72 h after the seizures and in adult rats (P60) [62].

In our previous study, we found that LTP attenuation in FS rats is due to a more pronounced desensitization of NMDA receptors. The application of D-serine, a co-agonist of the glycine site of NMDA receptors, allowed us to restore synaptic plasticity to the control value [25]. In this work, we obtained a similar result; in addition, we found that in P55 rats subjected to FSs D-serine was also efficient. It is known that astrocytes provide the local D-serine supply, thus changing NMDA-receptor-dependent signaling, and can enhance LTP [28]. The application of D-serine was shown to improve LTP in rats in the lithium–pilocarpine model. In the latent phase of the model, D-serine fully restored LTP in hippocampal slices [63], while in animals in the chronic phase, the effect was less pronounced and was only evident in the initial phase of LTP [64].

Due to the fact that FSs occur in early postnatal ontogenesis, when the processes of the maturation of neurons and astrocytes and the formation of synapses are active, some studies have raised the question of whether FSs lead to impairments in important processes such as learning and memory. Cognitive impairments after prolonged FSs have been described in both pediatric clinical studies [65] and several animal studies. In particular, adult rats subjected to FSs were found to have deficits in working and reference memory in the Morris water maze test and in long-term memory in a contextual fear conditioning test [45,53,66]. We also note that after prolonged FSs animals performed worse when we changed the escape box locations after several days of training in the Barnes maze. This result is similar to that previously described by Dube et al. using the Morris maze [1].

It should also be noted that the hippocampus is part of the limbic system and has connections with emotion-related brain regions, for instance, the amygdala and the prefrontal cortex. An impairment of synaptic plasticity in these areas of the CNS, resulting in an abnormal structural remodeling of these areas, may contribute to the pathophysiology of depression [67]. During early postnatal development, the hippocampus is highly susceptible to stress and other negative stimuli [68] and smaller hippocampal volumes have been reported in children and adults exposed to early-life adversity [69,70]. Although the nature of the associations among early-life adversity, hippocampal volume deficit, changes in neural plasticity, and depressive disorder are not well-understood, antidepressant treatment has been shown to protect against hippocampal volume loss [71,72] and affect the synaptic plasticity processes, in particular by increasing the expression of plasticity-related protein [73], and can relieve stress-induced inhibition LTP [74] in the hippocampus. Our data show that FSs lead to long-term LTP disorders in the hippocampus, and it was previously demonstrated that adult rats subjected to hyperthermia-induced seizures during the neonatal period demonstrate depressive-like behavior [75], which allows the consideration of antidepressant treatment as a potential method of correcting not only depressive behavior but also cognitive impairments that develop after FSs.

## 4. Materials and Methods

### 4.1. Animals

Wistar rats were used in this study. Animals were kept under standard conditions at room temperature with free access to water and food. All experiments were approved by the Sechenov Institute of Evolutionary Physiology and Biochemistry Ethics Committee and were carried out following local guidelines on the treatment of laboratory animals. These guidelines fully comply with Russian and international standards for animal studies.

### 4.2. FS Model

The nests were formed in such a way that each cage contained a female with 10 pups. This was to ensure that the animals from each litter had approximately the same body weight at P10.

Experimental FSs were induced at P10. The pups were placed on the bottom of a glass chamber for 30 min. At a height of 40 cm, warm airflow was created in such a way that a temperature of 46 °C was maintained at the bottom of the chamber, where the animal was located. Body temperature (rectal) was measured at baseline (31.1 ± 0.1 °C), at the onset of seizures (39.8 ± 0.1 °C), and every two minutes during seizures. If the body temperature was above 41 °C, the rats were transferred to a cool surface until the body temperature returned to 39 °C or less. The rats were then transferred back to the chamber. Under such conditions, the development and course of FSs in most animals were stereotypic: during the first 10 min, body temperature rose to 39 °C, and facial automatisms were observed, often accompanied by unilateral body flexion. Then, there were myoclonic twitches of the hind limbs, followed by clonic convulsions. Only animals with FSs that lasted at least 15 min were included in the study (*N* = 53). After hyperthermia, the animals were placed on a cold surface until the core temperature returned to the normal range. Then, they were returned to the home cage.

After hyperthermia, the pups’ weights changed insignificantly (<3% change in body weight), indicating only slight dehydration symptoms. The mortality rate during hyperthermia and the following 30 min was less than 1%.

Littermates used as controls were taken from the cage for the same time but were maintained at room temperature (*N* = 50).

### 4.3. Brain Slice Preparation

At P12, P21–23, and P51–55, the rats were decapitated, and brains were quickly removed. Horizontal brain slices (400 μm) were cut using an HM 650 V vibratome (Microm, Walldorf, Germany) in chilled artificial cerebrospinal fluid (ACSF; *t* = 0 °C; containing (in mM): 126 NaCl, 24 NaHCO_3_, 2.5 KCl, 2 CaCl_2_, 1.25 NaH_2_PO_4_, 1 MgSO_4_, and 10 glucose), which was aerated with carbogen (95% O_2_ and 5% CO_2_). Afterward, slices were allowed to recover at 35 °C in oxygenated ACSF for 1 h.

### 4.4. Field Potential Recordings

Extracellular field excitatory postsynaptic potentials (fEPSPs) were recorded from the CA1 stratum radiatum of the hippocampus with glass microelectrodes (0.2–1.0 MΩ). Synaptic responses were evoked by the stimulation of the Schaffer collaterals using a bipolar nichrome electrode as previously described [25]. Stimuli were delivered every 20 s via an A365 stimulus isolator (World Precision Instruments, Sarasota, FL, USA). Responses were amplified by a Model 1800 amplifier (A-M Systems, Carlsborg, WA, USA) then digitized with an ADC/DAC NI USB-6211 (National Instruments, Austin, TX, USA) using WinWCP v5.x.x software (University of Strathclyde, Glasgow, UK).

LTP was induced using high-frequency stimulation (HFS, three trains consisting of 100 pulses at 100 Hz applied every 20 s). A 20 min baseline period preceded LTP induction. Potentiated fEPSPs were recorded for 60 min following stimulation. LTP was quantified by calculating the ratio of the average slope of the potentiated fEPSPs (50–60 min after stimulation) and the baseline ones (10 min before stimulation). The recordings were analyzed using Clampfit 10.2 software (Axon Instruments, San Jose, CA, USA).

Dizocilpine (MK-801, 10 μM), a noncompetitive NMDA receptor antagonist, and D-serine, a co-agonist of NMDARs, were obtained from Sigma (St. Louis, MO, USA). These drugs were diluted in distilled water and bath-applied.

### 4.5. Immunohistochemistry

At P12 and P21–23, rats were deeply anesthetized with a mixture of Zoletil (3 mg per 100 g) and Xylazine (50 µL per 100 g) diluted in a saline solution and perfused transcardially with phosphate-buffered saline (PBS, pH 7.4, 0.01 M) followed by 4% paraformaldehyde in PBS. Next, decapitation, the extraction of the brain, and its fixation in 4% PFA at 4 °C for at least 24 h were performed. After fixation, brains were cryoprotected with 30% sucrose, frozen in cooled (<−50 °C) isopentane (78-78-4, Isopentane Solution, Sigma-Aldrich, St. Louis, MO, USA), and stored at −80 °C. The 20 μm-thick frontal serial sections (from −2.6 to −3.6 mm to the bregma) were cut on a Bright OTF5000 cryostat (Bright Instrument Co Ltd., Huntingdon, UK) and mounted on slides with the adhesive coating Super Frost Plus (J1800AMNZ, Fisher Scientific UK Ltd., Loughborough, UK).

The distribution of GFAP was analyzed using indirect immunofluorescence analysis. For blocking the endogenous peroxidase activities in the sections, 3% hydrogen peroxide was used for 30 min. After a rinse in PBS, sections were incubated in phosphate-buffered saline with 0.2% Triton X-100 (PBS-T) for 30 min then incubated in blocking serum (2% normal goat serum and 3% bovine serum albumin in PBS-T) for 1 h. Then, sections were incubated in blocking serum with the primary mouse antibody against GFAP (1:1000; cat # NBP1-05197, Bio-Techne Ltd., Abingdon, OX14 3NB, UK) for 48 h at 4 °C. Next, sections were incubated with a biotinylated goat antimouse secondary antibody (1:500 in PBS, cat # BA-9200-1.5, Vector Laboratories Inc, Burlingame, CA, USA) for 1 h and with streptavidin (1:1000 in PBS, cat # S2438-250UG, Sigma-Aldrich, St. Louis, MO, USA) for 1 h at room temperature. Then, sections were stained with 3,3’-diaminobenzidine (DAB), dehydrated, cleared, and permanently mounted.

At least 5–6 brain sections, taken at 100 μm intervals, were analyzed using the Leica AF 7000 Microscope (Leica Microsystems, Wetzlar, Germany) under ×400 magnification. The areas to be analyzed in each section were selected as shown in Figure 3a. The area (in %) occupied by GFAP-positive objects was assessed using ImageJ (U. S. National Institutes of Health, Bethesda, MD, USA). First, the images were converted to an 8-bit format. Next, we obtained the resulting image histograms representing the distribution of pixels from min–max (0–255) display values. The threshold option allowed us to choose a threshold value: pixels less than a certain value were recognized as one class (black), and pixels greater than the value were recognized as another class (white). The threshold value was determined individually for each image using the following protocol: (1) We found a value that included 99.5% of the pixels and multiplied it by 0.75. This value was considered the threshold value. (2) The GFAP-positive area was determined using the Analyze Particles tool (ImageJ). Objects with an area of less than 500 pixels were cut out of the final result using the Size function. Thus, for each image, the percentage of the area (%) occupied by GFAP-positive objects and a visual black-and-white mask were obtained.

### 4.6. Barnes Maze Task

The Barnes maze task was performed on P51–60 rats. The Barnes maze is a behavioral paradigm for assessing spatial learning and memory [76,77]. The apparatus used in the Barnes maze task included a circular white field (100 cm in diameter) with 20 circular holes (8 cm in diameter) evenly spaced around the periphery, an escape box (25 × 17 × 12.5 cm) for the animal to hide in installed under one of the holes, and a nontransparent box (diameter was 28 cm, and depth was 30 cm) for rat transportation. The field was located 80 cm above the floor, light sources brightly illuminated the field (illumination on the center of the field was 520 Lx), and a recording camera was placed above the field. The recordings were analyzed using the program “Pole Krest” designed at the Institute of Experimental Medicine for the analysis of animal behavior.

The experiment contained tree stages:

(1) Habituation: To familiarize the animal with the maze, habituation was performed 72 h before the beginning of the training. For this purpose, the rat was first placed in the escape box for 3 min. Then, this box was placed under the hole of a fully illuminated field. After that, the rat was placed in the center of the field. The escape box was under one of the holes, and the rat had to find it within 3 min. If during this time the rat did not find the escape box, it was gently pushed and forced to go down into the box, where it was left for 3 min. Then, the rat was placed in an opaque box, and the box was placed in the center of the field for 1 min. Then, the opaque box was removed, and the rat searched for the escape box again. The last step was repeated two times.
(2) Training: The location of the escape box was changed from the time of habituation and fixed during training. Training was performed over four consecutive days. The rat had two attempts per day. The rat was placed in the nontransparent box, and this box was placed in the center of the field for 1 min. After removing the nontransparent box, the recording began, and the rat had 5 min to find the escape box. If the rat did not find the escape box during this time, it was gently pushed to it and forced to go down into the box. After that, the rat remained in the escape box for 1 min. The second attempt began five minutes after the first attempt. Between each attempt, the field and box hole were cleaned with an alcohol–peroxide solution (0.3% peroxide and 30% alcohol). To assess progress in learning, we measured the time spent searching for the escape box.
(3) Testing (day 5): The day after the training was completed, the escape box was moved to another location in the opposite sector. On this day, the rat had one attempt to find the escape box. We measured the time spent in the target sector, where the box was located, during training.

### 4.7. Statistical Analysis

The statistical analysis was performed with OriginPro 8 (OriginLab Corporation, Northampton, MA, USA) and Statistica 8.0 (Systat Software Inc., Palo Alto, CA, USA). Dixon’s Q test (at a 90% confidence level) was used to identify and reject outliers. The normality of the sample data was evaluated using the Kolmogorov–Smirnov test. Statistical significance was assessed using Student’s *t*-test and one-way or two-way ANOVAs as stated in the text. All data are presented as means ± standard error of the mean. *p* < 0.05 was considered statistically significant.

## Data Availability

The data presented in this study are available on request from the corresponding author.
